# GOOD or BAD Responder? Behavioural and Neuroanatomical Markers of Clinical Response to Donepezil in Dementia

**DOI:** 10.3233/BEN-2011-0359

**Published:** 2012-01-20

**Authors:** Gabriella Bottini, Manuela Berlingeri, Stefania Basilico, Serena Passoni, Laura Danelli, Nadia Colombo, Maurizio Sberna, Massimo Franceschi, Roberto Sterzi, Eraldo Paulesu

**Affiliations:** ^1^Psychology DepartmentUniversity of PaviaPaviaItaly; ^2^Center of Cognitive NeuropsychologyNiguarda Cà Granda HospitalMilanItaly; ^3^Psychology DepartmentUniversity of Milano-BicoccaMilanItaly; ^4^Neuroradiology DepartmentNiguarda Cà Granda HospitalMilanItaly; ^5^Neurology UnitClinic "Santa Maria"CastellanzaItaly; ^6^Neurology DepartmentNiguarda Cà Granda HospitalMilanItaly; ^7^IRCCS GaleazziMilanItaly

**Keywords:** Acetilcholinesterase inhibitors, Alzheimer’s disease, donepezil, MRI, voxel-based morphometry

## Abstract

We explored the neuropsychological and neuromorphometrical differences between probable Alzheimer's disease patients showing a good or a bad response to nine months treatment with donepezil. Before treatment, the neuropsychological profile of the two patient groups was perfectly matched. By the ninth month after treatment, the *BAD-responders* showed a decline of the MMSE score together with a progressive impairment of executive functions. A voxel-based morphometry investigation (VBM), at the time of the second neuropsychological assessment, showed that the *BAD-responders* had larger grey and white matter atrophies involving the substantia innominata of Meynert bilaterally, the ventral part of caudate nuclei and the left uncinate fasciculus, brain areas belonging to the cholinergic pathways. A more widespread degeneration of the central cholinergic pathways may explain the lack of donepezil efficacy in those patients not responding to a treatment that operates on the grounds that some degree of endogeneous release of acetylcholine is still available.

### Abbreviations

| | |
|---|---|
| AAL | Automatic Anatomical Labelling |
| ACh | Acetilcholine |
| AChEIs | Acetilcholinesterase inhibitors |
| AD | Alzheimer's disease |
| ADL | Activity of Daily Living |
| cRPM | Raven's coloured Progressive Matrices |
| CSF | Cerebro-Spinal Fluid |
| FDR | False Discovery Rate |
| FWE | Family Wise Error |
| GM | Grey matter |
| IADL | Instrumental Activity of Daily Living |
| MMSE | Mini-Mental State Examination |
| VBM | Voxel-Based Morphometry |
| WhM | White matter |

### 1. Introduction

Alzheimer's disease (AD) is the most common degenerative neurological disorder, with a prevalence ranging from 1% of the 65 years old individuals to about 50% for the over-90s [1]. In particular, for a

ISSN 0953-4180/12/$27.50 © 2012 – IOS Press and the authors. All rights reserved country of about 60 million inhabitants like Italy, it has been estimated an incidence of 65000 new cases every year [2].

The cholinergic system is considered an important target of this disease to such an extent that a "cholinergic hypothesis" of AD has been widely explored since the early 70s [3,4]: the progressive degeneration of the basal cholinergic system and the resulting loss of cholinergic neurotransmission to the cortex is considered an important factor contributing to AD patients' cognitive deficits, a notion consistent with psychopharmacological studies which demonstrated an important role of ACh in memory and learning [see 5 for a recent review].

This evidence has justified the use of ACh-neurotransmission enhancing molecules in AD [6] including acetylcholinesterase inhibitors (AChEIs) which promote cholinergic neurotransmission by increasing the synaptic availability of ACh and which modulate other neurotransmitter systems as well [7–9].

Several clinical trials have supported the efficacy of acetylcholinesterase inhibitors (AChEIs) on the behavioural and neuropsychological manifestations of AD [10]. In particular, clinical longitudinal studies suggest that these drugs can delay the natural course of the worsening of the symptoms of about 1 year [11,12].

However, there are a number of factors that should be considered by the clinician when deciding either to start, or to continue, or to discontinue the AChEI therapy.

For example, the variability of the effect of AChEIs is rather large: a recent metanalysis showed that only the 33% of the patients treated with AChEIs are stabilized after 9 months [13]. The causes of this variability are currently unknown. In addition, according to Cochrane reviews [14–16], the effect over time of AChEIs is not a long-lasting one: in the *AD2000* study [17] the authors did not find any overall significant difference between AD patients treated with donepezil and AD patients treated with placebo after 9 months, the same time-window used in our study. However, it has to be pointed out that the lack of a significant effect in that study could have been due to the heterogeneity of the AD-treated patients as this group may have included both *BAD*- and *GOOD-responders* to the treatment.

Finally, economical considerations must also be taken into account: the cost of treatment with AChEIs may vary from 90 to 120 € per month per patient; the treatment may last up to 5 years, with a dropout of about 30% of cases in the first 6 months [17]. If one combines this information with the prevalence rate of the disease, the annual cost just for the drug may reach hundreds of million of Euros for a country like Italy [18].

Of course, if one could identify a predictive index of AD patients' response to AChEI treatments, the clinician can then optimize the prescription of these drugs. Several experimental strategies could be envisaged to address this issue: identification of biochemical, pharmacological indexes, in isolation or in combination, are among the possibilities. However, to date, there are no standard biochemical and/or pharmacological markers that could be used to diagnose AD in its early stages, nor to predict the efficacy of any treatment [19].

A possible complementary strategy could be the one of patient phenotyping through a combination of (functional)anatomical and behavioural measures: this approach should permit to identify, after a certain time-window, *GOOD*- rather than *BAD-responders* to the AChEI therapy.

Two recent SPECT studies investigated brain perfusion changes in AD patients during chronic AChEIs therapy in relation to their cognitive evolution [20,21]. In these studies, patients were classified as stable and not stable on the basis of their MMSE score change over time. The authors considered between-groups differences of regional cerebral blood flow (rCBF) as a neural index of the AChEIs effect on brain function. In both studies, a widespread reduction of rCBF was reported for the *BAD-responders*.

rCBF measurements in this endeavour have the obvious advantage of showing effects due to the actual degeneration of specific brain regions, together with functional effects on brain systems targeted by the degenerated brain areas. However, this advantage turns into a limitation if one wants to identify a causal relationship between regional effects and the response to the therapy as, on the grounds of rCBF measurements, it is impossible to distinguish rCBF decreases due to local neuronal degeneration from distant rCBF – diaschisis-like – decreases, due to dysfunctional effects associated with a deprivation of synaptic input. On the other hand, morphometric studies, like those based on voxel-based morphometry (VBM) [22,23], have the advantage to address the *barebones* of the pathology, at least at the level of description afforded by neuroimaging, the results not being confounded by functional-neurochemical effects.

To date, only one study, published in two separate papers by Venneri et al. [24,25], used VBM to address the issue discussed here. They reported the longitudinal grey [24] and white matter [25] volume changes in minimal-to-mild Alzheimer's disease patients receiving either rivastigmine (known to inhibit both butirrilcholinesterase and acetylcholinesterase) or more specific cholinesterase inhibitors, like donepezil or galantamine. They reported that the group of patients treated with rivastigmine had a smaller decrease of grey and white matter density over the 20 weeks of follow-up study. This interesting study showed the potentials of adopting a combination of behavioural and automated morphometric measures to monitor the response to specific treatments in AD.

The aim of our study was to further pursue this avenue. However, instead of comparing the effect of different kinds of cholinesterase inhibitors, we set ourselves to identify potential behavioural and anatomical markers of a clinical response to a widely used inhibitor of acetylcholinesterase: donepezil. To this end, a group of patients with a first diagnosis of probable AD, according to standard clinical criteria [26], was longitudinally studied over nine months; the response to donepezil was assessed by a measure of time related changes on the Mini-Mental State Examination test [MMSE; 27], an index of global cognitive function. This allowed us to distinguish *GOOD-* from *BAD-responders* and to evaluate in these two groups the neuropsychological and behavioural profile together with the morphological pattern of cerebral atrophy as studied with VBM.

### 2. Materials and methods

#### 2.1. Subjects and neuropsychological testing

Twenty-three consecutive patients with their first diagnosis of mild probable AD (MMSE score $\geqslant 18$), diagnosed according to the NINCDS-ADRDA criteria [26], and twenty-three normal controls participated in this study (see Table 1 for more details). The groups were matched for demographic variables (age and education).

The participants' cognitive profile was assessed using a neuropsychological battery investigating nonverbal reasoning [Raven Coloured Progressive Matrices; 28], selective attention [visual search test; 29], sustained attention [Trail Making Test, A; 30], verbal and spatial short-term memory [Digit Span task and Corsi's Block Tapping task; 31], verbal episodic memory [Memory for a Short Story; 29], language comprehension [Token Test; 29], controlled word retrieval [Phonemic and Semantic Verbal Fluency Tasks; 32]. Moreover, two behavioural scales were included in the battery: the Activity of Daily Living [ADL; 33], and the Instrumental Activity of Daily Living [IADL; 34]. Each patient was blood (BCB, azotemia, glicemya, calcemia, phosphorus, transaminase GOT and GPT, gammaGT, CPK, LDH, alkaline phosphatase, bilirubin, amylase, TSH, B12, folate), urine and ECG screened in order to exclude major contraindications to the start of AChEI therapy with donepezil within two weeks (T0). All the patients underwent also to a CT scan in order to exclude both significant vascular damages, with particular attention to white matter damages, both other neuroanatomical alterations.

Table 1
Demographic variables

| | *GOOD-responders* | *BAD-responders* | Elderly controls |
|---|---|---|---|
| N | 11 (6m/5f) | 12 (5m/7f) | 23 (11m/12f) |
| Age | 75.3 (6.2) | 76.1 (7.6) | 73 (4.9) |
| Educational level | 7 (4.9) | 6 (1.9) | 10.22 (4.4) |

After nine months from the diagnosis and from the beginning of the therapy (T1),[1] the AD patients were re-assessed with the same neuropsychological battery used at T0. The time-related change in the MMSE scores, namely the difference between the MMSE score at T0 and at T1, was then used as a global behavioural measure to classify the response to the treatment.

Because of the non-causal nature of the AChEI therapy in AD, we did not expect dramatic improvements of cognitive functioning, rather we assumed that a stable cognitive profile or a moderate improvement after nine months would have been a sign of a positive response to the treatment, counterbalancing the natural regression of the cognitive performance; on the other hand, a decline from T0 to T1 was interpreted as a sign of no response.

Following this rationale, the AD sample was divided in two sub-groups so that the patients whose time-related change of the MMSE score was greater than 3 points[2] were classified as "*BAD-responders*"

[1]The timing of the second assessment mimics the behavioral study in Courtney and colleagues [17]. The decision of making a single MRI scan at T1 was motivated by practical reasons, including financial constraints. We therefore decided to perform the single MRI scan at the time when we thought it could be maximal the chance of finding a morphometric difference between *GOOD-* and *BAD-responders*.

[2]This criterion was adopted on the basis of the results reported in the literature [35]. Moreover, in a post-hoc analysis, a reliable change (RC) index [36] was calculated on the entire AD sample. According to this analysis, a difference of 3 points between T0 and T1 at the MMSE corresponded to a RC = 1.12 which was equivalent to the 90% of probability of detecting a significant change.

Table 2
Neuropsychological data. Mean performance and Standard Deviations are reported for each neuropsychological test

| | T0 | | T1 | | T0-T1 | |
|---|---|---|---|---|---|---|
| | *GOOD-responders* | *BAD-responders* | *GOOD-responders* | *BAD-responders* | *GOOD-responders* | BAD-*responders* |
| MMSE | 22.82 (2.14) | 22.9 (2.35) | 24.18 (2.71) | **18.5**° (2.9) | −1.36 (2.73) | **4.41*** (2.1) |
| Phonemic fluency | 20.91 (10.34) | 20. 9 (5.45) | 21.64 (9.49) | **14.91**° (5.05) | −0.72 (5.79) | **6*** (3.64) |
| Semantic fluency | 10.32 (4.01) | 11.22 (2.4) | 9.8 (2.47) | 8.4 (2.33) | 0.52 (4.61) | **2.77*** (2.06) |
| Token Test | 28.85 (3.55) | 29.1 (2.2) | 30.73 (2.55) | **27.62**° (3) | −1.75 (3.83) | 1.16 (2.8) |
| Short Story Recall | 0.74 (1.12) | 1.17 (2.3) | 1.58 (2.25) | 2.1 (2.96) | −0.84 (1.94) | −0.99 (4.12) |
| Digit Span | 4.91 (1.38) | 4.9 (0.9) | 5.09 (1.22) | 4.75 (0.75) | −0.18 (0.87) | 0.16 (1.33) |
| Corsi's Span | 4.27 (0.65) | 4 (0.9) | 4.10 (0.57) | 3.5 (0.9) | 0.2 (0.63) | 0.41 (0.8) |
| Copy of Rey's Figure | 22.28 (8.71) | 22.85 (11.77) | 27.13 (6.51) | 19.79 (9.81) | −2.92 (9.23) | 0.85 (7.5) |
| Delayed Recall of Rey's Figure | 4.33 (5.21) | 0.1 (3.7) | 0.63 (1.77) | 0.58 (1.37) | 4.2 (4.77) | 0.14 (.37) |
| Visual Search | 36.27 (13.45) | 40.09 (8.74) | 35.5 (12.2) | 28 (10.59) | 1.1 (12.2) | **12.08*** (8.5) |
| Trail Making Test A | 129.09 (48.44) | 135.08 (52.47) | 140.7 (32.21) | 209.18 (106.74) | −13.7 (52.53) | **−83.63*** (94) |
| Raven's Coloured Progressive Matrices | 18.18 (7.04) | 20.27 (5.55) | 20.2 (3.49) | 16.16 (5.33) | −3 (6.23) | **3.54*** (5.6) |
| IADL | 6.09 (1.71) | 5.81 (1.47) | 4.60 (2.17) | 4.25 (1.8) | **1.4*** (1.89) | **1.82*** (2.48) |
| ADL | 6 (0) | 5.81 (0.4) | 5.30 (0.82) | 5.2 (1.54) | **0.7*** (.82) | 0.63 (4.5) |

*Within-group significant differences after the nine months of treatment calculated using the non-parametric Wilcoxon's Test.
°:Between-groups significant differences after nine months of treatment calculated using the non-parametric Mann-Whitney U Test.

(12 patients). The remaining patients were classified as "*GOOD-responders*" (11 patients). Following this classification, on average, the "*BAD-responders*" had a 4 point decline while, in fact, the *"GOOD-responders"* had a 2 point increase of the MMSE score, a between-group difference that, while not a dramatic one, was significant (see the results section and Table 2 for further details).

The time-related changes of the general cognitive level and of the various cognitive functions, assessed with the neuropsychological battery, were then evaluated through Wilcoxon signed-rank tests. Between-groups comparisons were performed using Mann-Whitney U tests.

It is worthy to note that at T0 all the patients were administered with a daily dose of 5 mg of donepezil – apart from one *GOOD-responder* patient who started with 10 mg. While after nine months of therapy (T1), the daily dose of donepezil was increased to 10 mg for all the patients excepted for a *GOOD-responder* patient that remains stable at 5 mg.

All participants gave their written consent to the experiment. The study was approved by the Ethics Committee of the Niguarda Cà Granda Hospital in Milan, Italy.

### 2.2. *Anatomical methods*

The AD patients (at T1) and the healthy elderly controls underwent also a Magnetic Resonance Imaging (MRI) scan.

MRI was performed on a 1.5 Tesla Marconi Philips Infinion echo-speed coil and amplifier hardware, using a standard head coil. A high-resolution, T1-weighted anatomical scan was acquired for each subject using a MPRAGE sequence (flip angle 35°, TE = 5 ms, TR = 21 ms, FOV = 256 × 192 mm, matrix 256 × 256, TI = 768 ms) with 140 axial slices (1 × 1 × 1 mm voxels).

Data were analysed on a Windows XP-PC workstation using Matlab 6.5 (MatWorks, Natick, MA, USA) and Statistical Parametric Mapping Software (SPM2, Wellcome Department of Imaging Neuroscience, London, UK, 2000).

MRI data were processed using an optimised VBM protocol, as described by Good et al. [23]. Accordingly, the entire brain volume was extracted from the native skull space to determine ideal stereotactic normalization parameters. In a second step, the native MRI scans were normalized to the stereotactic space and segmented into three different volumes: grey matter (GM), white matter (WhM) and CSF (cerebral-spinal fluid). A Jacobian modulation was applied to preserve an absolute regional amount of GM from the distortion introduced by the stereotactic normalization [22]. In the last step a spatial smoothing was performed using a Kernel Gaussian filter of 12, 12, 12 mm.

### 2.3. *Statistical analysis of the anatomical data*

The anatomical differences between the three groups were estimated with a series of t-test analyses on a voxel-by-voxel basis, while age and education were treated as confounding covariates. Moreover, in order to minimize the impact of inter-subject variability of global brain volume, regional values were first corrected using a proportional scaling technique.

Separate analyses were run for the GM and for the WhM data. We assessed differences of GM or WhM matter volume as (a) areas of shared atrophy in the two groups of patients as compared with the normal controls and (b) areas of tissue reduction in the *BAD-responders* as compared with the *GOOD-responders*.

Regional effects are reported at $p < 0.001$ (uncorrected); we also indicate whether a given region survived one of the two voxelwise corrections for multiple comparisons offered by SPM2, the Family-Wise Error correction (FWE) and the False Discovery Rate correction [FDR; 37].

Moreover, in order to explicitly test the GM differences in brain regions containing the body-cells of cholinergic neurons, a Region of Interest (ROI) approach was adopted. The ROIs corresponding to the basal forebrain structures, Meynert's nuclei, also known as "*substantia innominata*", were created by using the stereotactic coordinates reported in the paper by Teipel and colleagues [38] who studied the regional atrophy of these structures in Alzheimer's disease. For each coordinate, a sphere with 5 mm radius was designed using the software MRIcro: these served as an *inclusive mask* for the ROI oriented analysis on these regions. As these regions were selected on the basis of a strong a-priori hypothesis, the regional effects for the ROI analysis (i.e. the exploration of between-group differences in a restricted set of voxels in order to minimize the effect of multiple-comparisons) were assessed using an uncorrected $p < 0.05$ threshold.

Finally, using linear regression analyses, we evaluated to what extent GM or WhM volume correlated with behavioural measures used to assess executive functions, measures where *GOOD-* and *BAD-responders* differed at T1. This was done over the entire sample of AD patients.

The identification of the GM and WhM structures was based on the structural anatomical atlases available with the free-software MRIcron [39], in particular for the GM structures the AAL [Automatic Anatomical Labelling atlas; 40] was used, while for the WhM structures we referred to the JHU-White matter labels atlas [41].

### 3. Results

#### 3.1. Neuropsychological results

The raw MMSE score of the normal controls varied between 30 and 24 (mean score = 27.87; standard deviation = 1.36). When corrected according to Italian normative data [42] all the controls obtained a MMSE score higher than 26. Moreover, none of the controls obtained a pathological score in the other neuropsychological tests.

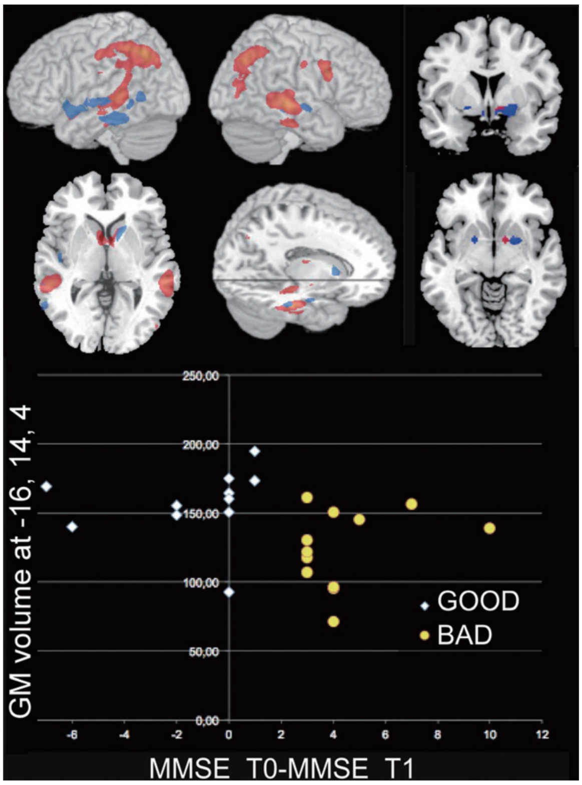

Fig. 1. GM volume reductions in *BAD-* and *GOOD-responders*. The red regions represent the GM volume reduction in the entire sample of AD patients when compared with healthy controls. The blue regions represent the brain area significantly more atrophic in the group of *BAD-responders* when compared with the *GOOD-responders*. The dispersion plot represents the level of GM volume in the left ventral caudate as a function of the time-related changes at the MMSE score, which suggests that the higher is the decline at the MMSE, the lower is the volume in the left caudate nuclei at T1 in the group of AD patients.

The MMSE score decrease over the whole sample of AD patients after 9 months was on average 1.6 (SD = 3.7). This finding is consistent with other data reported in the literature, for example those described by Cortes et al. [43] on a sample of unselected 686 patients studied during a similar time window.

Once the patients were classified as *GOOD-* or *BAD-responders* on the basis of the change of the MMSE score (see methods), we observed the following neuropsychological profiles.

At T0 the two groups of patients obtained a similar performance on the MMSE and on the other neuropsychological tests included in our neuropsychological assessment. Similarly, the two groups of AD patients did not differ in the behavioural scales used here (the ADL, the IADL). However, at T1, after nine months from the recruitment, some between-groups differences emerged. As expected, given the criterion used to split the AD patient sample in *GOOD-* or *BAD-responders*, the two groups now differed for the MMSE score (Mann-Whitney U test: $Z = -3.53$, $p < 0.0001$, see Table 2 for more details). At this time, there were also differences in language comprehension as measured by the Token Test – where the *GOOD-responders* mean score was 30.73 while the *BAD-responders* one was 27.62 (Mann-Whitney U test: $Z = -2.26$, $p < 0.03$; see Table 2 for more details) – and, at the Phonemic Fluency test – where the *GOOD-responders* were able to retrieve 21.64 words, while the *BAD-responders* retrieved only 14.91 words on average (Mann-Whitney U test: $Z = -1.98$, $p < 0.05$; see Table 2 for more details).

The within group time-related changes showed some additional interesting patterns. The *GOOD-responders* showed a decrement only at the ADL (Wilcoxon's test: $Z = -2.07$, $p < 0.04$) with a marginal tendency at the IADL (Wilcoxon's test: $Z = -1.9$, $p < 0.06$): the performance at the remaining tests was otherwise stable.

On the contrary, the neuropsychological profile of the *BAD-responders* significantly declined in several cognitive domains. In particular, the *BAD-responders* showed a significant score reduction at the MMSE (this was by definition our criterion for the identification of two patient groups, Wilcoxon's test: $Z = -3.1$, $p < 0.002$), at the Phonemic Fluency test (Wilcoxon's test: $Z = -3.06$, $p < 0.002$), at the Semantic Fluency test (Wilcoxon's test: $Z = -2.9$, $p < 0.003$), at the Visual Search test (Wilcoxon's test: $Z = -2.9$, $p < 0.003$), at the Trail Making test A (Wilcoxon's test: $Z = -2.05$, $p < 0.05$), at the cRPM (Wilcoxon's test: $Z = -2.2$, $p < 0.03$) and at the IADL (Mann-Whitney U test: $Z = -1.97$, $p < 0.05$).

A detailed report of the descriptive statistics for the neuropsychological scores is given in Table 2.

#### 3.2. Voxel-based morphometry results

##### 3.2.1. Between-groups comparisons: whole sample of AD patients versus controls

The comparison of the entire sample of patients with the normal controls showed the expected pattern of GM atrophy for AD patients [44,45]: this included the hippocampus and a large part of the temporal lobe bilaterally, the inferior frontal gyrus, and a diffuse parietal atrophy including the right inferior parietal lobule, the right angular gyrus and the right supramarginal gyrus and the postcentral gyrus bilaterally.

A significant GM volume reduction was also found in the basal ganglia, in particular in the caudate nuclei of both hemispheres, and in the left thalamus (see Fig. 1, areas in red, and Table 3 for more details). Finally, the results of the ROI analyses showed an overall bilateral reduction of the GM volume in Meynert's region.

The analyses of the WhM showed a significant reduction in the more extreme part of the uncinate fasciculi bilaterally, and in the WhM just below the precentral gyrus bilaterally, in the left limbic part of the internal capsule, in the left part of the WhM surrounding the thalamus and the striatum, in the WhM just below the left superior temporal gyrus, in the WhM adjacent to the left fusiform gyrus and to the right limbic areas (see Fig. 2, areas in red, and Table 3 for more details).

##### 3.2.2. Between-groups comparisons: BAD-versus GOOD-responders

Among the atrophic regions described above, the *BAD-responders* showed a significantly larger GM volume reduction in the Meynert's region, bilaterally, in the right insula, in the right superior temporal pole, in the superior temporal gyrus, in the middle and inferior temporal gyri, in the left putamen and in the left caudate nucleus, in the right pallidum and in the right caudate nuclei (see Fig. 1, areas in blue, and Table 3 for more details).

The *BAD-responders* also showed a greater WhM atrophy in the extreme part of the left uncinate fasciculus, in the left sagittal striatum fasciculus and in the WhM below the right precentral gyrus (see Fig. 2, areas in blue, and Table 3 for more details).

##### 3.2.3. Linear regression analysis

The linear regression analysis of the time-related differences between GM volume and the Phonemic Fluency test (T0-T1) showed a negative correlation with the regional GM volumes in those areas where the *GOOD-responders* and the *BAD-responders* differed in the between-group analysis. In particular, the time-related changes at this test significantly correlated with the GM volume in the left caudate nucleus, in the left temporal regions, in the left parahippocampal gyrus and in the insula bilaterally (Table 4 and Fig. 3).

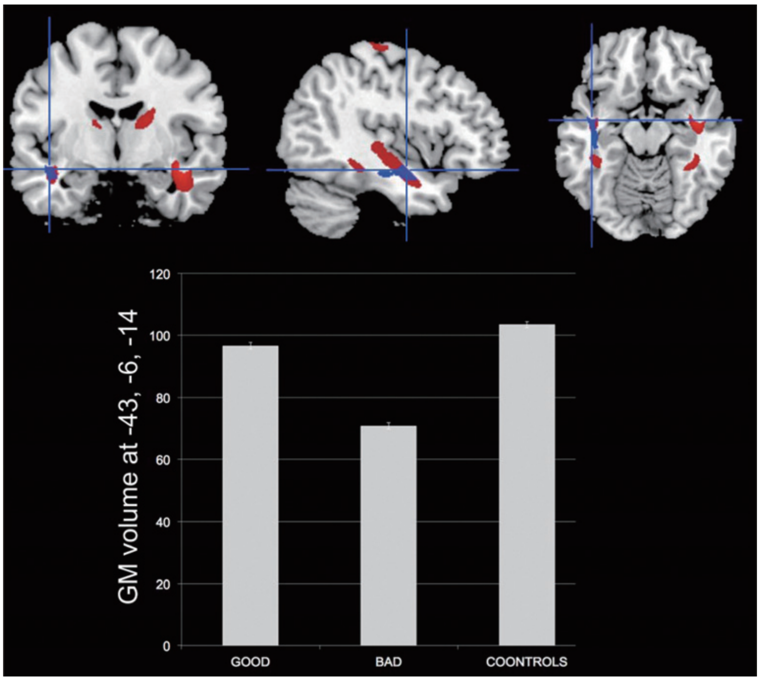

Fig. 2. WhM volume reductions in *BAD*- and *GOOD-responder*s. The red regions represent the WhM volume reduction in the entire sample of AD patients when compared with healthy controls. The blue regions represent the brain areas significantly more atrophic in the group of *BAD-responders* when compared with the *GOOD-responders*. The bar-graph represent the mean WhM volume extracted from the uncinate fasciculus. The error bars represent one standard deviation.

### 4. Discussion

Aim of this study was to explore in probable Alzheimer's disease patients the neuropsychological and neuromorphometric patterns associated with the variable response to a nine-month therapy with donepezil.

From a neuropsychological perspective, it is worth noting that our sample of AD patients, taken as a whole, was similar to those described in other studies concerned with the longitudinal changes of neuropsychological performance in AD. Indeed, the overall MMSE score decrease of our AD patients was very similar to the one recently described by Cortes et al. [43] over a similar time window [see Fig. 2 in 43].

The analysis of group-specific neuropsychological patterns of the patients, after their classification as *GOOD*- rather than *BAD-responders*, revealed some additional information. The two groups of patients did not show any behavioural difference at T0, rather their neuropsychological performance was comparable as much as they were the instrumental activities of daily living. At T1, however, some differences emerged: together with the MMSE score, whose changes were used as a classifying criterion to split the sample in *GOOD*- or *BAD-responders*, the two groups now differed also in a language comprehension task that implies the execution of increasingly complex verbal commands, the Token Test, and in a controlled word retrieval task measured by the frontal-lobe-tackling Phonemic fluency test [46]. These differences at T1 should most likely testify fairly different trajectories in the disease progression (see also Table 2, third column on *Time related changes*).

Clearly, behind the overall cognitive decline measured by a very popular but rather crude index like the MMSE, a more complex pattern is hidden. In particular, the tests where most of the *BAD-responders* showed a time-related decline were those tackling executive functions [47], a finding consistent with what is described as the typical progression of the disease [48–52]. Thus, although the use of the MMSE test to monitor the disease progression has the natural charm of the simplicity involved in both test administration and test scoring, it is clear that one could achieve a more detailed description of the neuropsychological trajectories associated with the disease by using a more extensive neuropsychological test battery.

On the other hand, the VBM results provide hints on the neuroanatomical underpinnings to the variable response to the therapy with donepezil. Our two groups

Table 4
Linear regression between Phonemic Fluency changes and GM volume in the entire sample of AD patients

| | MNI coordinates | | | | | | | |
|---|---|---|---|---|---|---|---|---|
| | *x* | *y* | *z* | *Z score* | *x* | *y* | *z* | *Z score* |
| Brain regions | Left hemisphere | | | | Right hemisphere | | | |
| Insula | −37 | 13 | −10 | 3.9# | 43 | −9 | −3 | 3.5 |
| | −40 | −13 | 1 | 3.1 | 43 | −6 | −8 | 3.4 |
| Olfactory cortex | −1 | 11 | −8 | 3.5 | | | | |
| Sup. temporal pole | −38 | 6 | −22 | 3.6 | | | | |
| | −39 | 10 | −18 | 3.5 | | | | |
| Sup. temporal gyrus | −46 | −10 | −12 | 3.7 | 44 | −11 | −7 | 3.5 |
| | −50 | −9 | −9 | 3.5 | | | | |
| Mid. temporal gyrus | −55 | −6 | −13 | 3.7 | | | | |
| | −46 | −6 | −14 | 3.7 | | | | |
| Inf. temporal gyrus | −40 | 8 | −41 | 3.1# | | | | |
| Parahippocampal gyrus | −19 | −22 | −18 | 4.7 | | | | |
| | −21 | 1 | −27 | 3.6 | | | | |
| Fusiform gyrus | −35 | −16 | −36 | 3.8 | | | | |
| Cerebellum | −49 | −56 | −52 | 3.7# | | | | |
| Cerebellum | −49 | −50 | −39 | 3.2# | | | | |
| Caudate nucleus | −9 | 15 | 2 | 3.3 | | | | |
| | −8 | 11 | 2 | 3.3 | | | | |

##= Correlation between GM volume and Phonemic fluency changes once the MMSE changes had been covariated out.

of patients showed the typical pattern of Alzheimer's disease-related GM atrophy in a relatively early-to moderate stage of the disease [45]: a significant reduction of the GM volume was found in the medial temporal structures bilaterally, in the basal ganglia bilaterally and in large part of the temporal and parietal neocortices. As expected, we did not find a pronounced frontal lobe atrophy, typically described in later stages of AD [53].

However, the AD patients not responding to AChEIs presented with a higher level of GM atrophy when compared with the *GOOD-responders*. This anatomical pattern included the ventral basal region of Meynert (substantia innominata), the ventral basal ganglia bilaterally and neocortical regions of the temporal lobe. In addition, we found a correlation between the progressive impairment of executive functions and the atrophy in the ventral part of the caudate nuclei.

The two groups of AD patients significantly differed also in the level of WhM volume in part of the left uncinate fasciculus and in the fiber blundles below the right precentral gyrus. It is worth of notice that most of these regions belong to a brain circuitry associated with ACh neurotransmission [54], including one major component, the basal forebrain region of Meynert and the so called "capsular division" of the ACh lateral pathway.[3]

These VBM data are in keeping with the behavioural patterns discussed above, and the two, taken together, are consistent with well documented correlations between the cholinergic circuitry, the basal ganglia and basal forebrain functions and their relationship with proficiency in tasks that depend on executive functions [55,56].

As the atrophy of the Ach-network was particularly prominent in the *BAD-responders*, our findings may provide an explanation on why part of AD patients do not show a response to the therapy with donepezil: we hypothesize that a severe and accelerated degeneration of the central cholinergic pathways might nullify the possibility of a sizeable clinical effect, as the treatment with acetylcholinesterase inhibitors operates on the grounds that some degree of endogenous release of acetylcholine is still available.

However, at the time of writing, it still remains to be explored whether the between-group morphometrical differences, identified at T1 on the basis of a differential response to donepezil, would be detectable at T0 al-

[3]The most part of the cholinergic pathways originates in the basal forebrain nuclei and in the amygdala. Starting from these structures, two different cholinergic pathways have been identified: (i) the medial pathway (supplying the olfactory, cingulate, pericingulate and retrosplenial cortices), and (ii) the lateral pathway which in turn is divided into a "*capsular division*" (travelling in the white matter of the external capsule, adjacent to the putamen and caudate nuclei, and uncinate fasciculus) and a "*perisylvian division*" (travelling within the claustrum) [54].

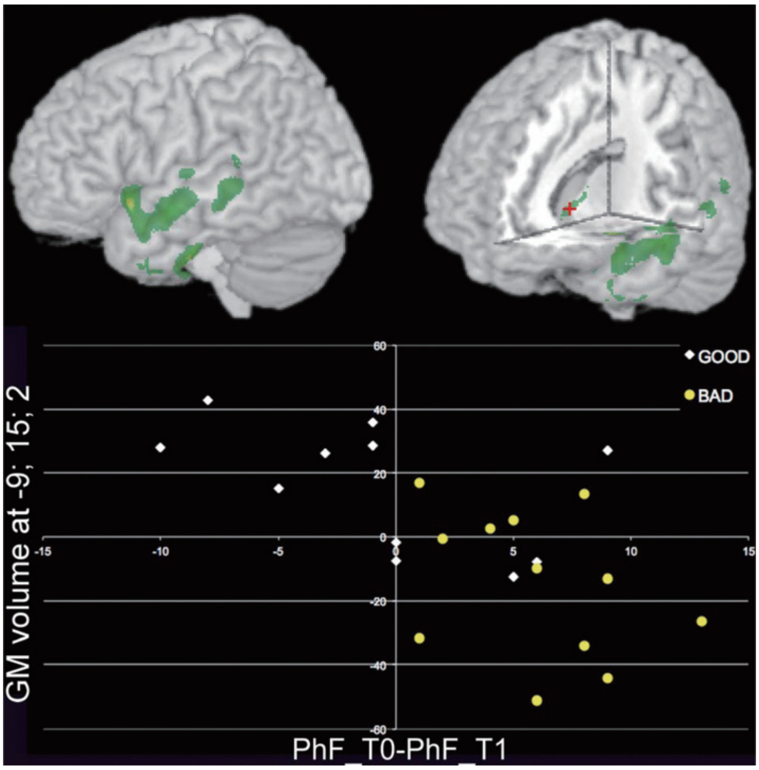

Fig. 3. Results from the linear regression analysis between changes at the Phonemic fluency test and GM volume. The green regions represent the brain area whose GM volume significantly correlate with time-related changes at the Phonemic fluency test (PhF_T0 – PhF_T1). The dispersion plot represents the distribution of GM volume extracted from the left caudate nucleus as a function of the decline of the performance at the Phonemic fluency test.

ready, or whether they would manifest themselves only after several months, nine in our case, together with a more pronounced neuropsychological decline. Previous data on MCI patients [57–59] and on the correlation between the MMSE score and the level of atrophy in different basal forebrain structures over-time [60] may suggest that our *GOOD-* and *BAD-responders* may have had similar atrophy at T0 while showing different trajectories of the disease progression over the nine months follow-up. However, only a longitudinal study with VBM may give an ultimate answer to this issue [61].

Another factor that remains unexplored by our study is the association between the apolipoprotein E (ApoE) genotype, the magnitude of the response to donepezil and the morphometric brain measurements. To date, the available evidence on this issue is contradictory, depending on the ApoE allele under consideration [24, 62,63].

Further studies, on larger samples, are needed to assess the several outstanding issues described before; however, our data suggest that the combined measure of cognitive functions, together with the morphometric measure of GM/WhM volume around the basal forebrain structures, may represent promising indexes to evaluate the response to AChEIs like donepezil in AD.

### Acknowledgments

This study was funded, in part, by a Grant from by Assessorato alla Sanità Regione Lombardia to Gabriella Bottini. Manuela Berlingeri was funded by the University of Milano-Bicocca and "Dote Ricercatori": FSE, Regione Lombardia, Italy.

Table 3
Voxel-based morphometry: between-groups comparisons

| | MNI coordinates | | | | | | | |
|---|---|---|---|---|---|---|---|---|
| | $x$ | $y$ | $z$ | Z score | $x$ | $y$ | $z$ | Z score |
| | Left hemisphere | | | | Right hemisphere | | | |
| **Grey Matter: AD < Controls** | | | | | | | | |
| Inf. frontal op. gyrus | −52 | 11 | 25 | 4.2° | | | | |
| Rolandic opercular gyrus | | | | | 47 | −18 | 24 | 3.4° |
| Postcentral gyrus | −58 | −12 | 35 | 3.5° | 50 | −17 | 41 | 3.9° |
| Supramarginal gyrus | | | | | 51 | −30 | 50 | 3.4° |
| Inf. parietal lobule | | | | | 44 | −53 | 46 | 4.3° |
| | | | | | 41 | −46 | 48 | 4.2° |
| Angular gyrus | −39 | −70 | 40 | 4.3° | 48 | −60 | 45 | 4.8*° |
| Precuneus | | | | | 10 | −70 | 41 | 3.7° |
| Sup. temporal pole | | | | | 42 | 9 | −18 | 3.6° |
| Sup. temporal gyrus | −63 | −27 | 7 | 3.7° | 49 | −28 | −2 | 3.9° |
| Mid. temporal gyrus | −56 | −29 | −3 | 4.7*° | 56 | −36 | 1 | 4.2° |
| | −62 | −40 | −3 | 4.0° | 53 | −31 | −2 | 4.0° |
| Inf. temporal gyrus | −43 | −28 | −19 | 4.1° | 47 | −26 | −26 | 3.6° |
| Fusiform gyrus | −37 | −26 | −16 | 3.8° | | | | |
| Parahippocampal gyrus | | | | | 24 | 8 | −32 | 3.3° |
| Hippocampus | −19 | −15 | −14 | 5.3*° | 36 | −26 | −11 | 4.2° |
| | −30 | −26 | −12 | 4.2° | 21 | −15 | −14 | 4.0° |
| Sup. occipital gyrus | | | | | 30 | −64 | 41 | 4.1° |
| Mid. occipital gyrus | −31 | −70 | 38 | 4.4° | 30 | −68 | 39 | 4.2° |
| Meynert's Basal nuclei | −4 | −2 | −10 | 1.8§ | 23 | −3 | −11 | 2.1§ |
| Caudate | −9 | 18 | 1 | 3.5° | 7 | 10 | 3 | 3.4° |
| Thalamus | −7 | −8 | 11 | 3.6° | | | | |
| **White Matter: AD < Controls.** | | | | | | | | |
| Sup. frontal WhM | | | | | 25 | 11 | 64 | 3.8 |
| Precentral WhM | −41 | −26 | 68 | 3.7 | 41 | −20 | 64 | 3.8 |
| Sup. corona Rad | | | | | 21 | −10 | 19 | 3.6 |
| Internal capsule | −11 | 0 | 11 | 3.7 | | | | |
| Sagital striatum | −39 | −38 | −16 | 3.4 | | | | |
| Post. thalamic WhM | −39 | −40 | 3 | 3.3 | | | | |
| Post. cingulate WhM | −13 | −48 | 20 | 3.5 | | | | |
| Ant. limbic WhM | | | | | 13 | 1 | 9 | 3.7 |
| Post. limbic WhM | | | | | 17 | −4 | −14 | 3.9 |
| Uncinate fasciculus | −41 | −4 | −16 | 4.1 | 38 | 0 | −25 | 4.4 |
| | | | | | 40 | −7 | −16 | 4.4 |
| Sup. temporal WhM | −44 | −26 | 0 | 3.4 | | | | |
| Fusiform WhM | −30 | −51 | −9 | 3.5 | 28 | −44 | −10 | 4 |
| | | | | | 36 | −39 | −15 | 3.8 |
| **Grey Matter: BAD < GOOD** | | | | | | | | |
| Insula | | | | | 39 | 16 | −8 | 3.8 |
| Sup. temporal pole | | | | | 43 | 11 | −14 | 4.0 |
| Sup. temporal gyrus | −48 | −11 | −7 | 3.4 | 46 | −11 | −5 | 4.3 |
| Mid. temporal gyrus | −51 | −29 | −3 | 3.5 | 62 | −58 | 0 | 3.7 |
| | −57 | −33 | −8 | 3.4 | 64 | −34 | −3 | 3.5 |
| Inf. temporal gyrus | | | | | 61 | −28 | −21 | 3.6 |
| Meynert's Basal nuclei | −18 | 8 | −6 | 1.9§ | 19 | 8 | −6 | 2.6§ |
| Putamen/Caudate | −16 | 14 | 4 | 4.0 | | | | |
| Pallidum/Caudate | | | | | 14 | 12 | −1 | 3.1 |
| **White Matter: BAD < GOOD** | | | | | | | | |
| Precentral WhM | | | | | 43 | −17 | 54 | 3.4 |
| Uncinate fasciculus | −43 | −6 | −14 | 3.6 | | | | |
| Sagital striatum | −41 | −22 | −15 | 3.4 | | | | |
| | −42 | −14 | −16 | 3.3 | | | | |

x, y, and z are the stereotactic coordinates of the activations in the MNI space. Statistical threshold $p < 0.001$ uncorrected.
*Z score statistically significant also after the FWE (Family-wise Error) correction.
°Z score statistically significant also after the FDR (False Discovery Rate) correction.
§Brain region identified using a ROI analysis on the basis of the coordinates proposed by Teipel et al. [35]. *GOOD* = *GOOD-responders* to donepezil, *BAD* = *BAD-responders* to donepezil.
